# Aneurism of the Brachial Artery Cured by Injection of Per Chloride of Iron

**Published:** 1857-06

**Authors:** M. LaGrange

**Affiliations:** Surgeon-in-Chief of the Civil and Military Hospital, at St. Mibriel, (Mense.)


### ARTICLE II.

From the "Gazette des Hospitaux."

TRANSLATED BY J. J. WEST, M. D. OF SAVANNAH, GA.

*Aneurism of the Brachial Artery cured by injection of per chloride of Iron.* By M. LaGrange, Surgeon in Chief of the Civil and Military Hospital, at St. Mibriel (Mense.)

Corporal Lacon, aged twenty-three years, sanguine temperament, belonging to the 5th regiment of Dragoons in garrison at Saint Mibriel, received in duel, a wound by the point of a sabre, which traversed horizontally, the thickness of the brachialis anticus muscle of the right arm, from before backwards, slightly from without inwards, (he being in gard and having the arm extended.)

The division of a small arterial branch gave place to a hemorrhage slightly abundant, which had as a consequence, an ecchymosis due to blood effused into the celbular tissue of the skin.

The small wound which this weapon occasioned appeared insignificant in regard to its depth, its diameter, and the parts interested; a simple dressing was prescribed, i. e. lotions of vegito—mineral water with a contentive bondage. A cure which seemed to be radical, was effected in eight days.

Five months later the Surgeon (" medicine major") of the regiment, at the request of the corporal whose duties had not been interrupted more than a week, saw his arm, in which a small tumour indolent had been progressively developed. He diagnosed an aneurism which decided him immediately to send the wounded to the hospital.

Upon his arrival 16th September, the tumour, situated upon the tract of the brochial artery of the right arm, at the superior third, 7 centimetres, (about three inches) from the cicatrix of the above mentioned wound, and bearing the form of a small egg, constituted an aneurism.

The information furnished upon its marble, upon the nature of the solution of the continuity of the muscle, and the examination of the member, and of the sabre, led to a discovery of its true cause in the lesion of the external or cellular arterial tunic superficially struck by the instrument, and in a gradual rupture of the two other tunics, abandoned to their own strengthand naturally pliable.

Yielding to the pressing solicitations of the patient, who desired to be promptly delivered of an affection, of which the gravity did not escape him. I resolved to satisfy him as speedily as possible, still not fixed upon a choice of means to adopt. The ligature of the brachial artery at its superior thiro, inspired me with real fears for the consequences of that operation. The injection of the per chloride of iron counts failures sufficiently striking to render circumspect and even fearful the most successful surgeons. It was necessary, however to decide for either one or the other of these two methods, because the compression indirect, generally employed at present in England, and which M. le docteur Broca proclaims in his "Traité des aneurisms," was not practicable in this case, and I confess that, having been sometimes a witness to sad terminations after the use of the ligature, my preferences drew me towards the injection of the per chloride of iron, the easy execution of which seduced me. I was curious to judge for myself of its veritable effects, and besides, I reflected, if the inflammatory accidents observed as results of this operation, can shake my determination, the happy facts produced by others ought to encourage me. I consulted two of my confriers, who, with me were in favor of employing the per chloride of iron.

The preparation marked 30° by the areometer of Baumé.

September 18, the patient seated upon a chair, having the arm in extension, the compression being confided to an aid I plunged a small trocar into the centre of the tumor from which a bright red blood escaped with considerable force. My intention was to disembarrass myself of it completely before making the injection; but at the moment when the solution penetrated, compression being no more properly exercised, the blood which filled the sac anew, coagulated instantaneously, and the artery which continued to pulsate, was not touched. The aneurism disappeared leaving in its place a hard insensible tumour, about the size of a nut. There was no pain except that of inflammation, and, eight days after, Lacon, exposed doubtlessly to a recurrence, left the hospital in excellent condition.

On the 14th October, this man, who had returned to his occupations, was sent back to me. Our previous apprehensions were realized; the aneurism rapidly reproduced in the superior part of the first sac, which retained considerable consistence, had acquired as great a developement as formerly. This time we took the minutest precautions to arrive with more certainty at our end.

The operation was performed on the 19th, in presence of Messrs. Laurens, professor in the School of Medicine of Maucy; Eraro, chief physician of the hospital, and Pougy, surgeon of the regiment. We again employed the solution indicated above. The compression established with care above and below the tumour, the same trocar was used, and the blood permitted to escape in totality. The injection was then made successively by ten or twelve drops at a time, until from forty to forty-five were thrown in, without the patient's experiencing the slightest impression. This amount reached, he gave utterance, all at once, to piercing cries, and complained of most atrocious pains in the axillary region, and the extremity of the little finger, caused by the irritating action of the solution on the median nerve. The arm reddened, became tumified with augmentation of head; retraction of the fore-arm. From three centimetres above to as far below the sac, the pulsations of the artery ceased, in all its extent marked by a hard cord, sensible and very appreciable to the touch. The radial and uluar arteries were no longer felt. The violence of the pain persisted during fifteen hours; the evening of the next day it was again very severe. The member, always swelled, preserved its heat.

From this time the inflammatory phenomena disappeared gradually under the influence of emollient applications, but the retraction of the fore-arm did not cease before the expiration of twelve days.

On the 25th October, feeble pulsations re-commenced in the uluar and became more and more pronounced; those of the anterior recurrent branch of the uluar had never been discontinued, but nothing palpable re-appeared in the brachial or at its bifurcation. It was not until the 17th November that pulsations began to be perceived by the touch. During ten days they were very obscure nothing being felt by the finger except a slight trembling. Little by little they augmented in force, without, however, arriving at a normal state. One month of repose was accorded the soldier, who perfectly cured, continues since that time, to enjoy the best health. He has recommenced his duties; the movements of the member are executed with ordinary freedom, and the elimination of the blood coagulated in the artery and the cyst has already reduced it sensibly in consistence and volume.

*Observations by the Translator.*—This method of treatment was conceived at thé commencement of the present century, but was not brought before the public as really practical, until three or four years since, when experiments were instituted by men whose names a failure could not injure, and whose efforts seemed for a while completely crowned with success. Their high authority with three brilliant successes, in a little time secured for it the warmest welcome, and it was at first believed about to supplant every other method of cure. The facility of its execution, with its apparent freedom from danger went greatly towards its general reception. We will see with how much justice these two great qualities were ascribed to it. The report which I have given above presents one of the dangers attending its use, that of consecutive inflammation, and the numerous failures to which it submitted, illustrates its facility. After the first cases were reported the whole profession grasped it with an energy only to be accounted for by the dangers attending, or almost perfect inutility of those methods then in use. The ligature, compression, direct or indirect, electro-puncture, were all objectionable, either by the danger and difficulty of the first, the difficulty and sometimes impossibility of the application and correct graduation of the second, and the general failure of the third. With all these considerations the profession was ripe for the reception of anything; the name of Lalleman heading as it did, the list was hardly necessary to warrant at least a trial. But contrary to the expectations and hopes of all, this new remedy was destined to sad reverses. The next cases in which it was tried were either partial or perfect failures. The old favorite, the ligature had in some, to be called in to its assistance and in others even that was not enough to prevent death. The reaction was complete, as well as sudden. The Society of Surgery condemned it, with hardly a dissenting voice. This was scarcely a year after its first appearance. But is it worthy this unanimous condemnation? or was it worthy the enthusiasm at first created in its favor? Perhaps neither the one or the other. It is hardly yet possible to appreciate its value. Its successes certainly seem to warrant its application, under some circumstances, and prove that it is not altogether a dangerous or useless remedy, while its reverses should make the surgeon careful in its use. To what cases is it applicable? under what circumstances should it be avoided? why does it succeed in one case and fail in another, seemingly analagous? These are questions yet to be answered. Some of these we will attempt in this article, others must be left for experiments yet to be instituted.

The credit of this invention is due to Monteggia, an Italian Surgeon, who first proposed the injection of astringents, such as alchol, taurine, acetate of lead, &c., into aneurismal sacs, for that very purpose, accomplished by the per chloride of iron, whose properties were not then known. (Istituzioni chirurgiche, (1813.) He refers to it in several parts of his work, and without laying too much stress on so new an idea, seems to regard it as not only plausible, but likely to succeed if properly attempted. He does not seem, however, to have experimented sufficiently to assert its superiority over other methods, but says enough to merit the praise of its invention, if not of its thorough application.

After him there was a long silence. In 1835 Mr. Leroy again brought it forward, not, however, in connection with the aneurismal sac directly, but rather to stop the flow of the blood in arteries. In his memoire he speaks of experiments made upon horses, carotid, and also in the use of the galvano-puncture. He found that the clot formed by the first was not sufficiently hard, and gave the preference to the latter. In 1841, Mr. Wardrop of London proposed the use of acetic acid; to him is due the idea of compression exercised on the artery above and below the tumor, (process carried out afterwards by Pravaz.) The use of sulphuric acid was proposed about the same time by Bouchut, but not attempted on account of its too caustic properties. Ramboud proposed and executed injections of vegetable acids, such as acetic and citric acids, and after a series of experiments gave the preference to the latter. His experiments were not made upon aneurisms, but upon other sanguine tumors, with which generally he succeeded, (1848).

Up to this time M. Pravaz of Lyons had been engaged in testing the coagulating property of his electricity. His purpose was that of many others, the cure of aneurisms, but when by the efforts of M. Petriquin this method had entered into common practice and its inefficacy sufficiently proved, his experiments in the laboratory brought to his mind the powerful effect of the per chloride of iron upon the blood, and he dreamed for the first time of its employment in aneurisms. He then recommenced the experiments of Leroy, but with a liquid incomparably superior. After having had constructed an instrument which still bears his name with that of Charrieri, he commenced with energy a series of trials which resulted in the most complete success. Before making much progress, however, he was interrupted by illness, and it was only at the urgent solicitations of Lallemahd, who comprehended fully their utility, that he consented to commence anew his experiments. Lallemaud, Pravaz and Petrequin then undertook together this important labor at the veterinary school of Lyons. They made injections into the carotids of sheep and horses, and so satisfactory were the results that Lallemaud immediately wrote to the Academy of Sciences to make known to his confriers, the precious discovery of Pravaz. This letter was inserted in the annual report of that institution, in 1852. After the death of Pravaz, in June 1853, Petrequin, with that bitterness and want of generosity to be found only among French physicians, accused Pravaz of desiring to assume the whole credit of the discovery and omitting altogether his name. Then followed a long and disagreeable personal discussion, which resulted happily in the confusion of Petrequin, and the honor of truth and the dead. The history of this subject shows that the principle, that of coagulating the blood in aneurisms, did not originate with Pravaz; for Monteggia, Bouchut, Leroy and Wardrop, with many others had been seriously engaged in the same pursuit, but Pravaz, and he only, deserves the credit of the introduction of the use of that fluid which has been found most successful. "Honor to whom honor is due."

Upon the return of Lallemaud to Paris, he communicated more fully to the Society of Surgery, the results of his experiments, and by a singular coincidence, M. R. Desloug-champs, on the same day made a report of a case of aneurism, cured by two injections of the per chloride of iron. This was a traumatic aneurism of the *supra orbital artery.* The history of the case given above, is much the analogue of that reported by M. D—s, the ultimate result, the cure, being the same. M. Nipce, reported another, April 25, which was also successful, but, in which the accidents were much more severe; the consecutive inflammation existed for many days, and on the eleventh day of the tumor, which fluctuated, had to be opened, giving exit to a considerable quantity of a sero purulent matter. On the 20th day the cure was complete, nothing being felt in the place of the aneurism but a small, hard and indolent tumor.

Another case with still severer accidents was reported the 9th of May by M. Serres. The tumor was an aneurismal varix of the end of the elbow; coagulation was obtained as in the other cases, and when the compression had been removed pulsation had entirely ceased. The radial and ulnar continued to pulsate, but the pulsations ceased in them also, after the clot, by progressive formation had passed the point of junction of the recurrent branches. Intense inflammation was developed, which only yielded after the pus formed in the sac had been evacuated. An eschar was formed and was detached without hemorrhage.

These three cases were the first reported after the discovery of the influence of the per chloride of iron, and although they all resulted favorably, Mr. Malgaigue remarked that the accidents were severer progressively from the first to the third. After these, others were published in different parts of the country, giving various results from complete cure to the saddest end. Without enumerating the whole of these, or entering into a minute account of any of them, I will merely give the results of a few. The first reverse was sustained by a Mr. X of Paris, who neglected to make sufficient compression: the result was gangrene which necessitated amputation, and the patient died. Three others lives were lost, and several times the patient could be saved, only by the timely application of the ligature. These accidents warranted Mr. Malgaigue in reading before the Academy a memoire against its use, which struck this new method a blow, from which it has not yet recovered. Three new successes obtained in a few weeks, were not enough to efface this impression, so strong is the authority of the Academy. Was the sentence just? Could not this method be employed in America with more success? In the first place almost all of these experiments, for we can thus name them, were made in hospitals, either in Paris or the provinces, and the average mortality consequent on severe operations, is so much greater in the hospitals of Europe than in private practice, that we can hardly judge of the efficacy of this method by the cases which have been reported thus far. Secondly, I find by looking carefully over the list, that in several of the disastrous applications, the most necessary provisions were entirely omitted or not properly observed, such as compression above and below, the injection of too much of the liquid or of a preparation so strong as to become a caustic to the tissues; its use in cases altogether hopeless from the situation of the aneurism ; the adjustment ot an injurious apparatus to effect compression after the injection; and perhaps, an after treatment, not well directed. Still however, there were cases, in which all was done that could have been, and yet, important operations were rendered necessary, and even death supervened in spite of all the skill ot the first surgeons in the world. Twenty-one cases in all were reported, the comparative reiults of which do not seem favorable to this method. Omitting one of the Aorta, another of the Arteria Innominata, and still another of the sub-clavian Artery; of the remaining 18 there were nine cures, four deaths, and five cases necessitating severe surgical operations.

There is still another consideration not recommending too strongly this new method ; it was this, that in almost all cases in which it has been employed with success, the tumor was so situated that the ligature might have also been applied with perhaps less danger. One of the necessary features of those cases permitting the injection of this fluid, also permits the ligature, viz: the possibility of compression's being exercised above the tumor. In no case where compression was not or could not have been made, was the treatment successful; and this compression must be *perfect;* now where this is possible what hinders a skillful surgeon's trying the artery, when he is comparatively free from the terrible dangers of the consecutive inflammation and gangrene.

Without pursuing these remarks any further, I will give a brief and general review ot the modus operandi, for the benefit of those of your readers who have not read the admirable treatise of M. Broca, on aneurisms. To do this I will follow his remarks as closely as the brevity of this article will allow.

The best substance for injection is that which unites the three following conditions: 1st prompt coagulation, 2d, the formation of a clot of great solidity, 3d. action on the tissues but slightly irritated. Unhappily the last seems incompatible with the others. The chloride of zinc, taurine, alcohol, creasote and the vegetable acids fail in one or more of these qualities. Thus far the per chloride of iron seems to unite them more perfectly than any other substance known, for that reason, it is the one generally employed. All the persalts of this metal possess coagulating properties, but the others are too irritating to be made use of. The irritating principle of this fluid is said to exist in its chlorine, and apparently with justice, as it is known that the action upon the tissues is in proportion to the strength of the solution. The acetate of iron has been employed in two cases with success, by Mr. M. Lussana and Pavesi, but has not been sufficiently experimented with to give satisfactory results.

Experience has proved, that the strength of the solution of the per chloride of iron best adapted to this purpose is about 30° areometer of Baumè. Higher than that figure, it acts as a caustic, producing destruction of the tissues, and in many cases gangrene. A still lower is perhaps safer, but when carried below 15° a 20°, the action is slow and very imperfect; the clot that is formed is soft, and acts perhaps more as a foreign body than when the tissues are sufficiently irritated to throw out an adhesive and organizable plastic lymph.

A graduated syringe, with the piston grooved so as to turn like a screw, and to inject a known quantity with each turn, is the instrument of Pravaz. Each half-turn injects about one drop, a measurement sufficiently exact. A small trocar and canula fitting precisely the extremity of the syringe is also necessary for the completion of the apparatus. For the operation, the first movement is to secure the compression of the artery, which must always be made *below* the tumor, first. That above had better be made by the tourniquet for greater security, as the danger of a flow of the substance in the direction of the larger arteries is always more to be feared. Compression being satisfactorily adjusted, the trocar is plunged into the tumor, and then withdrawn, leaving the canula well in the cavity: the syringe is then fixed securely into the free extremity of the canula, and the handle is turned by degrees, waiting a few seconds after each four or five drops injected, until a clot is formed in the aneurism and the artery adjoining. Compression should be exercised during five or ten minutes after the formation of the clot, to prevent a flow of blood against the newly formed clot, or an entrance of the injected fluid farther than is absolutely necessary. When about to withdraw the canula, it is best to give a turn of the instrument backward: this action fills the extremity of the canula by a clot, and prevents the contact of the liquid still in its cavity with the tissues, covering the tumor. It has been seen that extreme pain and consecutive inflammation are almost always consequences of this operation; to combat them, the usual anodyne and antiptilogistic treatment, sometimes pushed with vigor, are necessary. Nothing acts better than the cold water dressing so common with us.

